# Supplementary material for: Investigating the quality of HIV rapid testing practices in public antenatal health care facilities, South Africa
Source: PLoS One. 2022 Aug 29;17(8):e0268687. doi: 10.1371/journal.pone.0268687 (PMC9423613; doi:10.1371/journal.pone.0268687)
Supplement: S1 Table — (DOCX) [file pone.0268687.s001.docx]

# Supporting information

**S1 Table: Distribution of facilities according to province, sentinel site and locality type in South Africa**

|  | Number of facilities* | % |
| --- | --- | --- |
| Province |  |  |
| Eastern Cape | 43 | 12.4 |
| Free State | 17 | 4.9 |
| Gauteng | 81 | 23.3 |
| KwaZulu-Natal | 77 | 22.1 |
| Limpopo | 43 | 12.4 |
| Mpumalanga | 29 | 8.3 |
| North West | 20 | 5.7 |
| Northern Cape | 8 | 2.3 |
| Western Cape | 30 | 8.6 |
| TOTAL | **348** | **100** |
| Sentinel site |  |  |
| ANSUR | 217 | 62.4 |
| NON-ANSUR | 131 | 37.6 |
| TOTAL | **348** | **100** |
| Locality type |  |  |
| Rural | 183 | 52.6 |
| Urban | 165 | 47.4 |
| Total | **348** | **100** |
