# Supplementary material for: Investigating the quality of HIV rapid testing practices in public antenatal health care facilities, South Africa
Source: PLoS One. 2022 Aug 29;17(8):e0268687. doi: 10.1371/journal.pone.0268687 (PMC9423613; doi:10.1371/journal.pone.0268687)
Supplement: Attachment — Submitted filename: RESPONSE To REVIEWERS _PLOSONE_07April2022 .docx [file pone.0268687.s003.docx]

**Reviewer’s Comments PONE-D-22-00472**

**Reviewer** # **2**

1. The study was conducted in 2017. Are there any changes in National HIV strategy since then? Are the findings relevant in 2022?

***Response:*** *Response: The findings are still relevant in 2022. South Africa is still implementing the 2017-2022 National HIV Strategic Plan aims at an intensified focus on districts and locations with high burdens of HIV, STIs and/or TB; on adolescent girls and young women and on tailoring interventions for the key and vulnerable populations.*

2. Elaborate RTCQI in Abstract section as it is not a common abbreviation (Line 24).

***Response:*** *The full name for RTCQI is ‘Rapid Test Continuous Quality Improvement. It has been added in the abstract on page 5 (line 24).*

3. Is it “Countries” or “Districts”? (Line 93).

***Response****: The word countries has been replaced with districts under Introduction, on page 8 (line 96)*

4. Statistical difference (P value) among the Provinces can be stated for better understanding. (Table 2)

***Response:*** *statistical significance test (p-value) has been added under results, page 13 Table 2 (lines 205-6)*

5. Conclusion section should contain detailed recommendations on the basis of study observations. Moreover, based on study limitations, suggestions can be made for future research studies for generating more generalizable and valid findings.

***Response****: The conclusion section has been revised. Detailed recommendations based on study limitations have been made on pages 26 (lines 365-378)*

***Specific feedback:***

**Reviewer #1:**

1.The Introduction part can be reduced, it is bit lengthy. Although the matter of introduction is relevant, but it can be concised.

***Response****: The introduction has been reduced as suggested from 1 050 to 851 words (pages 5-8).*

2. The Sampling strategy may be elaborated: what was sampling unit, how the sample size calculated, how strata's were defined and selected and finally how were the facilities selected.
Inclusion and exclusion criteria may be included.

***Response****: The sampling strategy has been revised under methods (page 9 (lines 114-140)*

*:*
