# Supplementary material for: Investigating the quality of HIV rapid testing practices in public antenatal health care facilities, South Africa
Source: PLoS One. 2022 Aug 29;17(8):e0268687. doi: 10.1371/journal.pone.0268687 (PMC9423613; doi:10.1371/journal.pone.0268687)
Supplement: Attachment — Submitted filename: PONE-D-22-00472 Reviewer Comments.docx [file pone.0268687.s002.docx]

**Reviewer’s Comments PONE-D-22-00472**

1. The study was conducted in 2017. Are there any changes in National HIV strategy since then? Are the findings relevant in 2022?
2. Elaborate RTCQI in Abstract section as it is not a common abbreviation (Line 24).
3. Is it “Countries” or “Districts”? (Line 93)
4. Statistical difference (P value) among the Provinces can be stated for better understanding. (Table 2)
5. Conclusion section should contain detailed recommendations on the basis of study observations. Moreover, based on study limitations, suggestions can be made for future research studies for generating more generalizable and valid findings.
